# Microarray expression profiling of *fndc3a* zebrafish mutants

**DOI:** 10.17912/micropub.biology.000646

**Published:** 2022-09-30

**Authors:** Daniel Liedtke, Eva Klopocki

**Affiliations:** 1 Institute of Human Genetics, Julius-Maximilians-Universität Würzburg, 97074 Würzburg, Germany

## Abstract

The group of Fibronectin type III domain-containing (FNDC; InterPro IPR003961) protein super family splits into a large number of gene-orthologues and mediates a variety of cellular functions during development and disease. They act as anti-inflammatory factors, are linked to cell-cell-interactions, regulate cell signaling and are associated with different cancer types, like cervical and colorectal. One member of this gene family is *FNDC3A* , which influences different developmental processes in vertebrates, like Sertoli cell/spermatid adhesion in mice testis, bone traits in chicken, and fin development in zebrafish. To identify downstream molecular processes during vertebrate development we investigated gene expression profiles in the previously established *fndc3a* zebrafish mutants via microarray analyses on 22 hpf embryos (26-somite stage). Our analyses imply distinct transcriptional profiles between genotype groups and hint to altered cell binding and catalytic activity in *fndc3a* mutants.

**Figure 1. Analysis of microarray expression profiles of zebrafish fndc3a mutants at 26-somite stage:**
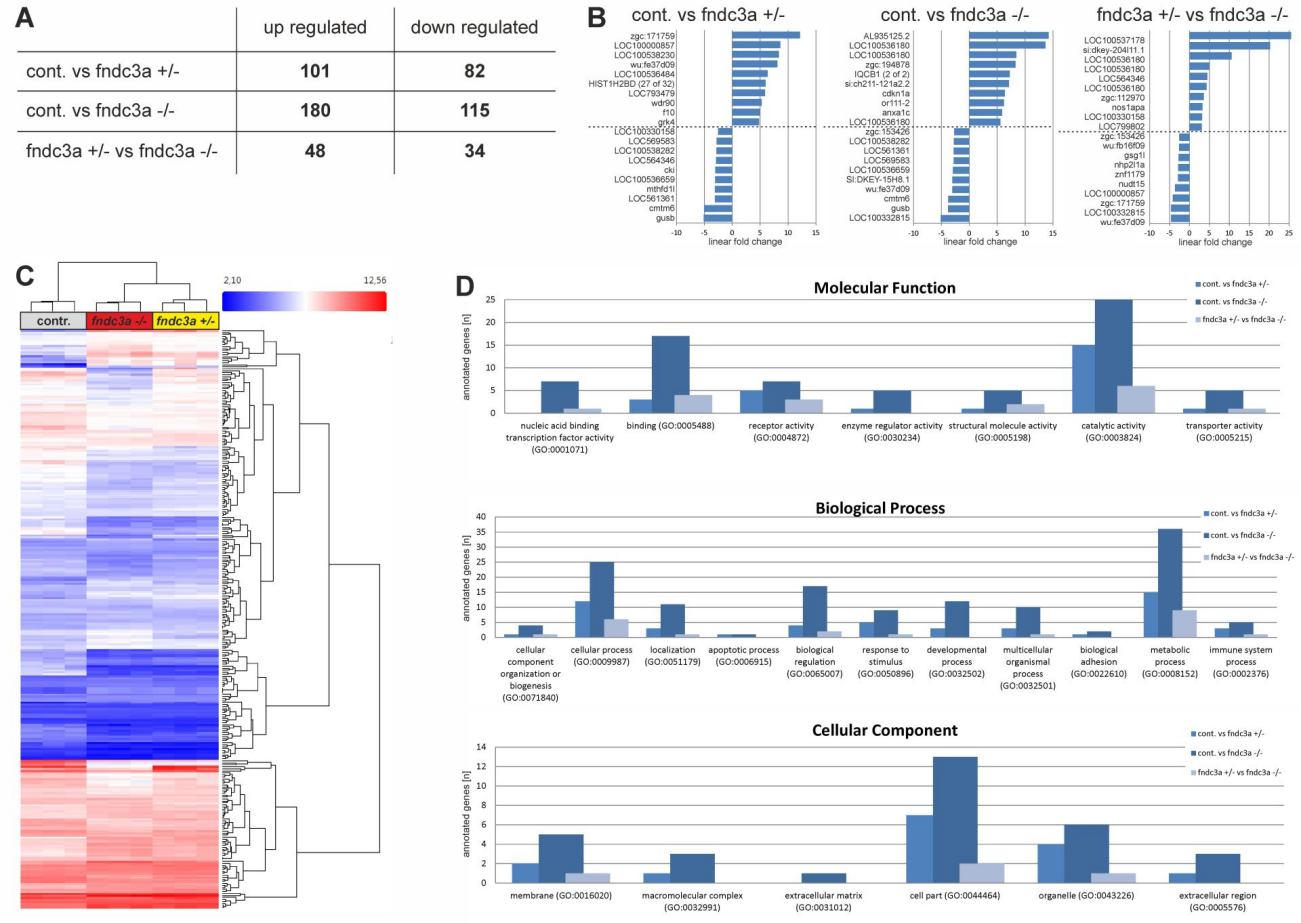
**A)** Number of up and down regulated transcripts in different genotype groups of *fndc3a* mutants. Comparison between different genotypes via Gene Level Differential Expression Analysis. **B)** Top 10 up- or downregulated transcripts in different genotype comparison groups. Given values are linear fold changes. **C)** Hierarchical clustering of transcripts comparing all nine experimental samples via Gene Level Differential Expression Analysis. Colors indicate fold changes, p value < 0.001. **D)** Panther GO term analyses of genotype comparison groups with focus of molecular function, biological processes and cellular components. Values imply number of up or down regulated genes for each condition and GO term (Go IDs; PANTHER 10.0 database). cont.: *fndc3a ^+/+^* wildtype embryos, *AB* strain; fndc3a +/-: *fndc3a ^wue1/+^* heterozygous embryos; fndc3a -/-: *fndc3a ^wue1/wue1^* homozygous embryos.

## Description

Fndc3a is one member of the FNDC-super family and contains nine fibronectin type III domains. These fibronectin type III domains are a common feature of a large number of extracellular proteins and are evolutionary conserved in a large number of species. Molecular studies have shown *FNDC3A* expression in human odontoblasts (Carrouel et al 2008). Functional experiments in *Symplastic spermatids* ( *sys* ) knockout mice indicated that Fndc3a is essential for cell adhesion between spermatids and Sertoli cells, resulting in sterile males after depletion (Obholz et al, 2006). Our previous work has shown that interference with Fndc3a function in zebrafish ( *Danio rerio* ) CRISPR/Cas9 mutants results in defects during tail bud development and caudal fin regeneration (Liedtke et al, 2019). The purpose of this follow-up study was to investigate potential downstream targets of Fndc3a during zebrafish development.

The established *fndc3a* CRISPR mutant line displays a 5 bp indel alteration in exon 13 (GRCz11 Chr.15: 3018433 – 3018438; genomic feature *wue1* ), resulting in potential frameshift and a premature STOP codon. The corresponding molecular analyses clarified a hypomorphic and temperature-sensitive phenotype in the generated *fndc3a* line, although homozygous embryos display a prominent tail fin phenotype during development and caudal fin regeneration defects (Liedtke et al, 2019). Earliest developmental changes in the ventral tail bud region of homozygous mutants was observed at 21-somite stage (19.5 hours post fertilization (hpf)).

To further investigate early transcriptional and developmental alterations in the *fndc3a* mutant line we performed Affymetrix microarray analyses. Whole-RNA was extracted from pools of 26-somite stage embryos (22 hpf) of different genotypes and was subsequently used for Gene Level Differential Expression Analysis. Each genetic condition was analyzed in biological triplicates and three independent embryo pools for each genotype were hybridized to Affymetrix Zebrafish Gene 1.0 ST Arrays. Unbiased post-run analyses showed clustering of genotypes and significant differences between genotype groups. Further detailed comparison between different genotype conditions indicated many up and down regulated transcripts (Fig. 1A and 1C). Besides a large number of unresolved or ill-described transcripts (Fig. 1B; e.g. *gusb* , zgc:175127, LOC100332815, AL935125.2), a number of genes could be identified which have been previously linked to embryonic development in zebrafish (see suppl. table 1). These genes have been described to be essential for development of cells located in the tail bud region or show expression within the tail bud surrounding tissues at this stage and are thereby in accordance with the investigated developmental stage and the previously observed *fndc3a* mutant phenotype. For example, *anxa1a* which is involved in caudal fin regeneration shows a reduced expression in homozygous mutants (fold change -2.77; contr. vs. homo; Saxena et al, 2016, Rabinowitz et al, 2017), thus, matching with caudal fin regeneration defects observed in these mutants. In addition, *gata1a* is a transcription factor involved in hemopoiesis and is overexpressed in blood precursor cells at this stage (fold change 2.53; contr. vs. homo; Lyons, et al 2002). These cells are partly located in the blood island area, which is affected by ECM defects in ventral fin fold tissues of *fndc3a* mutants. A last example is overexpression of *vent* (fold change 3.25; contr. vs. homo) *,* a homeobox gene which is embedded in a regulatory network along with *vox* to repress dorsal cell fates during tail bud development in zebrafish (Imai et al, 2001). To identify generally affected molecular functions, biological processes and cellular components in the mutants we performed additional GO term analyses (PANTHER database). These analyses indicate that reduction or loss of Fndc3a function predominantly affects transcripts linked to binding and catalytic activity, and to cellular and metabolic processes (Fig. 1D). Future studies will have to elucidate functions of ill-described transcripts and will have to link Fndc3a function to well-described genes during early tail bud development in zebrafish.

## Methods

*1) RNA extraction and microarray analyses:* RNA extraction of three genetic genotype groups (cont., het., homo.), each group consisting of 12 pooled embryos without chorion, has been performed with QIAzol lysis reagent according to RNeasy whole RNA extraction protocol (QIAGEN). Quality control of total RNA extraction via Eukaryote Total RNA Nano assay on a Bioanalyser (Agilent) and only RNA samples with RIN values larger than 5 were used for subsequent cDNA synthesis, fluorescent labeling and microarray hybridization. Quantification of transcriptional changes in mutants was done by Affymetrix microarray analyses using “Zebrafish Gene 1.0 ST Array” and Affymetrix software packages (Affymetrix). Microarray experiments were performed in cooperation with Core Unit Systems Medicine Würzburg according to Affymetrix specifications.

*2) Data analyses:* Data analyses has been performed via Affymetrix Transcriptome Analysis Console software. GO term analyses were performed with PANTHER classification system version 10.0 ( http://www.pantherdb.org/ ). Figure data and graphics have been assembled with Excel (Microsoft Corporation), OriginPro (OriginLab Corporation), and CorelDraw Graphics Suite (Corel Corporation). Original microarray data files are deposited in ArrayExpress (Accession number E-MTAB-11958, release date 01. August 2022) and can be downloaded at https://www.ebi.ac.uk/arrayexpress/experiments/E-MTAB-11958 .

*3) Animal handling:* All procedures involving experimental animals were performed in compliance with German animal welfare laws, guidelines, and policies. Generation of the used *fndc3a* mutant strain and founder animal genotyping via fin clipping was approved by the Committee on the Ethics of Animal Experiments of the University of Würzburg and the corresponding legislative body (Regierung von Unterfranken; Permit Number: DMS-2532-2-13 and DMS-2532-2-9). The generated *fndc3a* mutant line has been previously described and is submitted to ZFIN.org (Liedtke et al. 2019; in-house line ID: *fndc3a-41/5* ; genomic feature: *wue1* ). Zebrafish embryos used for this study were raised at 28.5°C in Danieau´s medium (17.4 mM NaCl,0.21 mM KCl, 0.12 mM MgSO4, 0.18 mM Ca(NO3)2, 1.5 mM Hepes, pH 7.2, 0.001% phenol blue) and were staged according to Kimmel at al 1995.

*4) Statistical analysis:* Statistical comparison of different genotype groups were performed with the following settings via Affymetrix Transcriptome Analysis Console software: One-Way Between-Subject ANOVA (unpaired); Fold Change (linear) < -2 or Fold Change (linear) > 2; ANOVA p-value (Condition pair) < 0.05; Triplicate conditions: wild type, *fndc3a* het and *fndc3a* homo. Gene Level Differential Expression Analysis with this settings (data shown in Fig. 1 and in Supl Data Fig1) indicated overall 405 differentially expressed genes from a total number of 59384 analyzed genes.

## Reagents

**Table:** 

**Strain**	**Genotype and ZFIN ID**	**Source**
* fndc3a ^wue1^*	Chr.15: 3018433 - 3018438 (GRCz11), CATCA/TTCTC; ZDB-ALT-170417-3	established CRISPR mutant line, previously published (Liedtke et al, 2019)

## Extended Data

Description: Extended excel table summarizing presented microarray data and analyses.. Resource Type: Dataset. DOI: 10.22002/yn220-zt682
